# Magnetic Graphene Oxide for Dual Targeted Delivery of Doxorubicin and Photothermal Therapy

**DOI:** 10.3390/nano8040193

**Published:** 2018-03-27

**Authors:** Yu-Jen Lu, Pin-Yi Lin, Pei-Han Huang, Chang-Yi Kuo, K.T. Shalumon, Mao-Yu Chen, Jyh-Ping Chen

**Affiliations:** 1 Department of Neurosurgery, Chang Gung Memorial Hospital Linkuo Medical Center and College of Medicine, Chang Gung University, Taoyuan 33305, Taiwan; alexlu0416@gmail.com (Y.-J.L.); giselle.huang@gmail.com (P.-H.H.); mailtomaxi@gmail.com (M.-Y.C.); 2 Department of Chemical and Materials Engineering, Chang Gung University, Taoyuan 33302, Taiwan; arrow06280@hotmail.com (P.-Y.L.); onesky1997@gmail.com (C.-Y.K.); shalumon@gmail.com (K.T.S.); 3 Department of Plastic and Reconstructive Surgery and Craniofacial Research Center, Chang Gung Memorial Hospital, Linkou, Kwei-San, Taoyuan 33305, Taiwan; 4 Research Center for Food and Cosmetic Safety, Research Center for Chinese Herbal Medicine, College of Human Ecology, Chang Gung University of Science and Technology, Kwei-San, Taoyuan 33302, Taiwan; 5 Department of Materials Engineering, Ming Chi University of Technology, Tai-Shan, New Taipei City 24301, Taiwan

**Keywords:** graphene oxide, magnetic nanoparticles, doxorubicin, cetuximab, photothermal therapy

## Abstract

To develop a pH-sensitive dual targeting magnetic nanocarrier for chemo-phototherapy in cancer treatment, we prepared magnetic graphene oxide (MGO) by depositing Fe_3_O_4_ magnetic nanoparticles on graphene oxide (GO) through chemical co-precipitation. MGO was modified with polyethylene glycol (PEG) and cetuximab (CET, an epidermal growth factor receptor (EGFR) monoclonal antibody) to obtain MGO-PEG-CET. Since EGFR was highly expressed on the tumor cell surface, MGO-PEG-CET was used for dual targeted delivery an anticancer drug doxorubicin (DOX). The physico-chemical properties of MGO-PEG-CET were fully characterized by dynamic light scattering, transmission electron microscopy, X-ray diffraction, Fourier transform Infrared spectroscopy, thermogravimetric analysis, and superconducting quantum interference device. Drug loading experiments revealed that DOX adsorption followed the Langmuir isotherm with a maximal drug loading capacity of 6.35 mg/mg, while DOX release was pH-dependent with more DOX released at pH 5.5 than pH 7.4. Using quantum-dots labeled nanocarriers and confocal microscopy, intracellular uptakes of MGO-PEG-CET by high EGFR-expressing CT-26 murine colorectal cells was confirmed to be more efficient than MGO. This cellular uptake could be inhibited by pre-incubation with CET, which confirmed the receptor-mediated endocytosis of MGO-PEG-CET. Magnetic targeted killing of CT-26 was demonstrated in vitro through magnetic guidance of MGO-PEG-CET/DOX, while the photothermal effect could be confirmed in vivo and in vitro after exposure of MGO-PEG-CET to near-infrared (NIR) laser light. In addition, the biocompatibility tests indicated MGO-PEG-CET showed no cytotoxicity toward fibroblasts and elicited minimum hemolysis. In vitro cytotoxicity tests showed the half maximal inhibitory concentration (IC50) value of MGO-PEG-CET/DOX toward CT-26 cells was 1.48 µg/mL, which was lower than that of MGO-PEG/DOX (2.64 µg/mL). The IC50 value could be further reduced to 1.17 µg/mL after combining with photothermal therapy by NIR laser light exposure. Using subcutaneously implanted CT-26 cells in BALB/c mice, in vivo anti-tumor studies indicated the relative tumor volumes at day 14 were 12.1 for control (normal saline), 10.1 for DOX, 9.5 for MGO-PEG-CET/DOX, 5.8 for MGO-PEG-CET/DOX + magnet, and 0.42 for MGO-PEG-CET/DOX + magnet + laser. Therefore, the dual targeting MGO-PEG-CET/DOX could be suggested as an effective drug delivery system for anticancer therapy, which showed a 29-fold increase in therapeutic efficacy compared with control by combining chemotherapy with photothermal therapy.

## 1. Introduction

Claiming the lives of 8.8 million people in 2015 alone, cancer is always a serious leading cause of death worldwide [1]. Currently, there are several different treatment techniques, including surgery, radiation, chemotherapy, targeted therapy, and immunotherapy [2]. Among these, chemotherapy has remained as one of the most common therapy methods for the treatment of different kinds of cancers. However, to be successful, chemotherapy may be dependent on several factors, including optimization of drug delivery to a specific targeting site, hence minimizing undesirable side effects to normal cells [3].

Advanced drug delivery systems are able to overcome the problems in conventional chemotherapy by offering carrier systems the possibility to hold sufficient amount of drug, prolong the circulation time, and provide controlled release of drug within tumor cells [4]. In particular, the application of nanotechnology in chemotherapeutics has huge potential to overcome the problems faced in drug delivery, and also provides a platform for the development of a multi-functional drug delivery nano-system for theranostic nanomedicine [5]. Although several nanomaterial-based chemotherapeutics have been successfully translated to clinical applications, the successful clinical translation of promising nanotherapy from benchside to bedside still faces plenty of hurdles. The inconsistency between the pre-clinical and clinical studies and the heterogeneity found in tumors may be suggested as two of the major challenges that nanomaterial-based anti-tumor therapies are facing for translational medicine [6].

Nanoparticles provide ample means of enforcing targeted therapy via passive targeting that refers to efficient localization of nanoparticles within the tumor microenvironment, as well as active targeting that represents the active uptake of nanoparticles by tumor cells. The miniscule size of nanoparticles not only enables far greater intracellular uptake as compared with micron-sized particles [7], it also allows for an inherent passive targeting by means of the enhanced permeability and retention (EPR) effect across tumor tissue’s leaky microvasculature [8,9,10]. Furthermore, new classes of carbon-based nanomaterials, such as carbon nanotube [11] and graphene [12], augment the passive targeting mechanism by releasing their therapeutic moieties in response to a given external environment pH. As epidermal growth factor receptor (EGFR) is highly expressed on the surface of tumor cells, active targeting could be achieved through the surface modification of nanoparticles with a targeting ligand, the EGFR monoclonal antibody (cetuximab, CET), to increase the bindings of drug-loaded nanocarriers with surface receptors of cancer cells and to significantly enhance their intracellular uptake by targeted cancer cells [13,14]. On the other hand, there have been numerous studies exploring the conjugation of magnetic nanoparticles with chemotherapeutic agents, which can then be specifically targeted to localized tumors by guidance with an external magnetic field, hence further increasing the efficiency of anti-cancer therapy through magnetic targeting [15,16,17].

Graphene is currently the thinnest material in existence with a thickness of only 0.35 nm [18]. It has a two-dimensional planar structure composed of a sp^2^ mixed-layer orbital with a considerably large specific surface area, making it suitable for carrying large quantities of substances (e.g., metal, biomolecules, and drugs) [19]. When used as a drug carrier, graphene is typically converted into graphene oxide (GO) to increase hydrophilicity by the introduction of oxygen-containing functional groups and bind with chemotherapeutic drugs, such as doxorubicin (DOX), by physical adsorption [20,21]. With loading capacities of up to 2.35 mg/mg of DOX, GO has a substantially greater loading capacity than other conventional drug carriers, such as polymeric micelle, hydrogel microparticles, and liposomes [22]. Furthermore, the adsorption between GO and DOX is pH-sensitive, which offers controlled drug release after intracellular uptake of DOX-loaded GO by cancer cells through endocytosis into the endosomes for release of its cargo in the low pH (~5) endosomal environment. Indeed, the blood’s physiological pH (pH 7.4) is expected to prevent burst DOX release in the circulation after intravenous injection of DOX-loaded GO, whereas the lower pH environment after endocytosis into cancer cells would trigger the release of the drug intracellularly for enhanced chemotherapeutic efficacy [22,23,24].

It should be noted that current literature proposes that GO may induce the generation of reactive oxygen species in target cancer cells, which was deemed as one of the most important nanotoxicity mechanisms of GO [25]. Nonetheless, the nanotoxicity depends on the number of layers, size, surface properties, and methods of the synthesis of GO, in addition to the dose, time of exposure, cell type, and administration method [26]. Thus, generalizations of GO nanotoxicity should be avoided due to the presence of several parameters affecting the toxicity profile of GO. For cellular responses to sheet-like GO, discrepancies were reported for different cell types [27]. However, GO of greatly different sizes (350 nm vs. 2200 mm) was selectively internalized by two macrophages by phagocytosis and showed equal uptake amount in macrophages [28].

Another advantage that is offered by GO is its strong optical absorption in the near-infrared (NIR) tissue transparency window that may allow its potential use as a photothermal therapy agent. Photothermal therapy involves the use of light absorbents so as to absorb 808 nm NIR light and convert the light energy into thermal energy for the killing of cancer cells and the ablation of tumor tissue [29,30]. In recent years, researchers have successfully demonstrated that GO exposed to NIR could destroy cancer cells in vitro and shrink tumor size from animal experiments [31]. Thus, irradiating GO with NIR light after its intracellular uptake by cancer cells could be employed as a noninvasive method for cancer treatment in conjunction with its advantages as a nanocarrier for DOX.

Taken together, all of the considerations mentioned above, we aim to utilize GO’s unique properties, pH-sensitive drug release, high drug loading, and strong optical NIR absorption, to develop a multi-functional DOX-carrying drug delivery system that incorporates dual-targeting drug delivery with photothermal therapy. We focus on using magnetic graphene oxide (MGO) by chemical co-precipitation of Fe_3_O_4_ magnetic nanoparticles on GO nano-platelets [32], which was further modified with polyethylene (PEG) and CET (MGO-PEG-CET), for magnetic and the receptor-mediated dual targeted delivery of DOX. We thoroughly characterize the properties of the nanocarriers and evaluate the anti-cancer therapeutic efficacy both in vitro and in vivo using high EGFR-expressing CT-26 murine colorectal cells.

## 2. Results and Discussion

### 2.1. Preparation and Characteriazation of MGO, MGO-PEG-CET and MGO-PEG-CET/DOX

**Figure 1:**
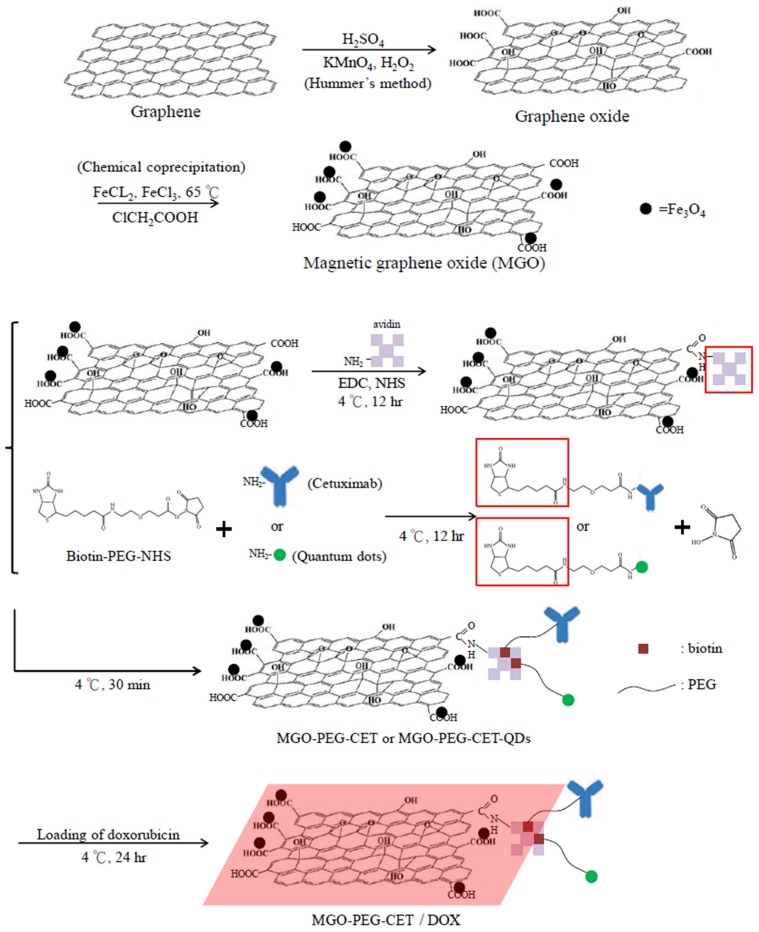
The flow diagram for producing doxorubicin (DOX)-loaded Magnetic Graphene Oxide (MGO)-polyethylene glycol (PEG)-cetuximab (CET) (MGO-PEG-CET/DOX). Fe_3_O_4_ magnetic nanoparticles were deposited on GO by chemical co-precipitation to prepare MGO. Avidin was bound to MGO by covalent binding while biotin-PEG N-hydroxysuccinimide (NHS) was conjugated to cetuximab (CET) or quantum dots (QDs). Mixing of avidin-modified MGO with biotin-PEG-CET and biotin-PEG-QDs could produce MGO-PEG-CET and MGO-PEG-CET-QDs for doxorubicin (DOX) loading and fluorescent tracking after intracellular uptake.

The nanocarriers were synthesized according to the scheme in Figure 1. Modification using ClCH_2_COOH introduced abundant –COOH groups on MGO surface, which reacted with the –NH_2_ groups of avidin through carbodiimide-mediated amide bond formation catalyzed by 1-(3-dimethylaminopropyl)-3-ethylcarbodiimide hydrochloride (EDC) and *N*-hydroxysuccinimide (NHS). Using biotin-PEG-NHS that has a terminal –NHS groups to react spontaneously with the –NH_2_ groups of CET (or quantum dots, QDs), we prepared biotinylated PEG-CET (or PEG-QDs). Finally, MGO-PEG-CET (or MGO-PEG-CET-QDs) could be facially prepared by taking advantage of the high affinity of avidin toward biotin (*K_d_* = 10^−15^ M) and the capacity to bind up to four biotin molecules per avidin [33]. Also, as the bond formation between avidin and biotin is very rapid and is not unaffected by pH and temperature, which further facilitate the approach adopted here to conjugate CET to PEGylated MGO.

This method also provides a simple method for PEGylation of MGO, which is expected to decrease the aggregation of MGO by diminishing its interaction with serum proteins to modulate the EPR effect, reduce the reticuloendothelial system (RES) uptake, and increase the circulation time of MGO-PEG [34]. It was demonstrated that after coating with PEG, drug-loaded nanocarriers could accumulate less in the liver to result in higher tumor accumulation than unmodified ones without PEGylation [35]. From chemical analysis, the toluidine blue O (TBO) dye adsorption assay confirmed that each milligram of MGO contained 2.38 × 10^−4^ ± 5.04 × 10^−5^ mmol of –COOH for conjugation with avidin. Quantitative analysis with protein assays indicated that the amount of avidin and CET conjugated to MGO is 1.938 ± 0.102 mg of avidin and 2.52 ± 0.212 mg of CET per mg of MGO, respectively.

**Figure 2:**
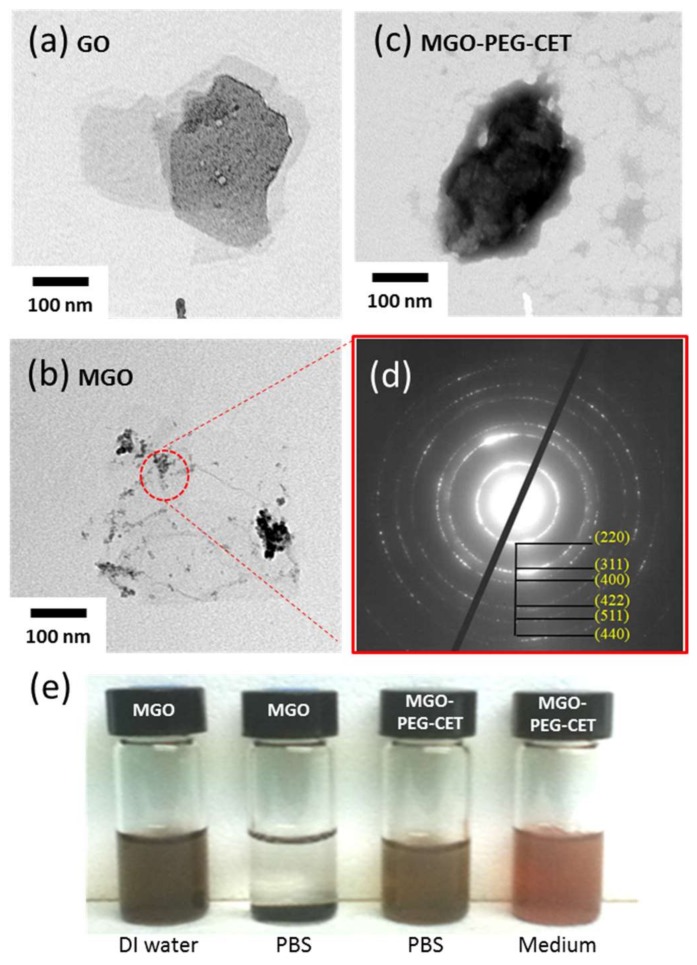
Transmission electron images of graphene oxide (GO) (**a**); MGO (**b**); and, MGO-PEG-CET after staining with 2% phosphotungstic acid (**c**); (**d**) The selected area electron diffraction patterns of MGO in the circled area in (**b**); (**e**) The suspension stability of 0.1 mg/mL MGO and MGO-PEG-CET in deionized (DI) water, phosphate buffered saline (PBS) and cell culture medium after 24 h.

The structure of GO, MGO and MGO-PEG-CET were observed under transmission election microscope (TEM). Figure 2a shows the laminar stacking form of GO with size less than 300 nm, while Figure 2b depicts the appearance of black nanoparticles in MGO from the electron dense magnetite after chemical co-precipitation of Fe_3_O_4_ magnetic nanoparticles. After negative staining with 2% phosphotungstic acid that selectively binds to the basic groups (lysine and arginine residues) of proteins [36], magnetic nanoparticles appeared black while the light grey zones revealed the presence of avidin and CET in the TEM image of MGO-PEG-CET (Figure 2c). Figure 2d illustrates the crystal lattices of Fe_3_O_4_ on the MGO detected through selected area electron diffraction of the circled area of MGO, which further confirms the presence of well-crystallized Fe_3_O_4_ magnetic nanoparticles in MGO. For suspension stability, 1% MGO is stable in deionized (DI) water for 24 h, but not in phosphate buffered saline (PBS) (Figure 2e). Consistent with a previous report that surface modification with PEG and proteins (avidin and CET) could substantially improve the stability of GO [37], MGO-PEG-PET was confirmed to be free from agglomeration and sedimentation in PBS and cell culture medium after 24 h (Figure 2e).

Form Table 1, the average hydrodynamic diameter obtained from dynamic light scattering (DLS) indicated that the particle size was significantly increased from GO to MGO after decorating GO with Fe_3_O_4_. However, the particle size showed no significant difference between MGO and MGO-PEG-CET after grafting with soft polymer chains. As nanoparticles with hydrophilic surfaces and threshold size ~200 nm will show improved EPR effect by increasing residence time in blood [38], MGO-PEG-CET falls within this size range and is expected to reach the tumor and endocytosed by cancer cells. For zeta potentials, GO showed a highly negative value due to the presence of abundant oxygen-containing functional groups on GO surface. The zeta potential of MGO slightly increased to −35.1 mV as Fe_3_O_4_ showed a positive zeta potential (28.8 ± 0.3 mV) from the –NH_2_ groups in ammonia used during chemical co-precipitation. When CET was conjugated to MGO, the zeta potential of MGO-PEG-CET further increased to ~20 mV as CET is a chimeric antibody with an isoelectric point of about 8.5 [39]. Nonetheless, the zeta potential is still high enough to confer dispersion stability by resisting aggregation.

**Figure 3:**
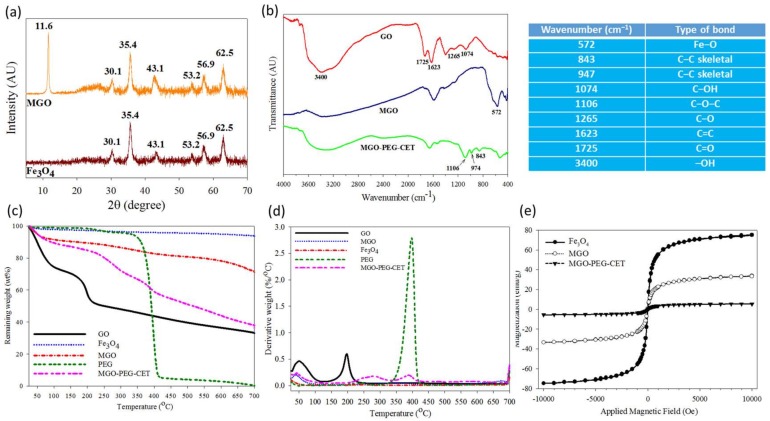
Characterization of MGO and MGO-PEG-CET by X-ray diffraction (XRD) (**a**); Fourier transform infrared (FTIR) (**b**); thermogravimetric analysis (TGA) (**c**); differential thermal analysis (DTA) (**d**); and superconducting quantum interference device (SQUID) (**e**). The table lists the wavenumbers of characteristic peak found in the FTIR spectra in (**b**).

Figure 3a shows X-ray diffraction (XRD) patterns of Fe_3_O_4_ and MGO. GO will show a typical diffraction peak at 2θ = 11.6° due to the (0 0 2) reflection with spacing d = 0.91 nm [40,41]. For MGO, new diffraction peaks were found at 2θ = 30.1°, 35.4°, 43.1°, 53.2°, 56.9°, and 62.5°, and identified as the cubic spinel crystal planes of Fe_3_O_4_ from JCPDS database [42]. In addition, the crystallite size of Fe_3_O_4_ could be estimated to be 10.9 nm from the strongest (3 1 1) reflection using the Debye-Scherrer equation [43].

From Fourier transform infrared (FTIR) analysis in Figure 3b, GO reveals characteristic bands at 1265 and 1074 cm^−1^ due to C–OH and C–O stretching vibrations, while the characteristic bands at 1623 and 3400 cm^−1^ are due to C=C and –OH [44]. In addition, the band at 1725 cm^−1^ could be assigned to C=O stretching vibrations from carbonyl and carboxylic groups in GO. MGO shows an additional characteristic peak at 572 cm^−1^ from the stretching vibration of Fe–O bond, suggesting that Fe_3_O_4_ is bound to GO successfully. For MGO-PEG-CET, additional peaks at 843, 947, and 1106 cm^−1^ could be assigned to PEG [45].

Thermogravimetric analysis (TGA) was conducted on GO, Fe_3_O_4_, MGO, and MGO-PEG-CET (Figure 3c). After initial weight loss due to water, substantial weight loss (~21%) was shown by GO when heated from 130 to 250 °C with the decomposition of its oxygen-containing functional groups, which gave a peak decomposition temperature of 200 °C and 33% residual weight at 700 °C [46,47]. Fe_3_O_4_ had minimal weight loss of ~3% at 700 °C, corresponding to the loss of surface –OH functional groups [48]. PEG underwent ~90% weight loss between 330 °C and 420 °C with a peak decomposition temperature at ~400 °C from the differential thermal analysis (DTA) curve in Figure 3d, to give zero residual weight at 700 °C, as expected for an organic polymer [49]. For MGO, thermal decomposition was delayed and more residual weight (~72%) than GO was found at 700 °C. The difference in the residual weight between GO and MGO was used to calculate the mass percentage of Fe_3_O_4_ in MGO, which was ~42% after considering the weight loss of Fe_3_O_4_. When considering the TGA curve of PEG, MGO-PEG-CET showed additional weight loss from 340 to 440 °C and a peak decomposition temperature at ~400 °C, which correspond to the thermal decomposition of PEG and confirms the presence of PEG in the nanocarrier. The final residual weight was also consistent with MGO-PEG-CET (38%) < MGO (72%), as grafted PEG, in addition to avidin and CET, will contribute to additional weight loss. Using inductively coupled plasma-optical emission spectroscopy (ICP-OES), the weight percentage of Fe_3_O_4_ in MGO could be also confirmed to be 43%.

A superconducting quantum interference device (SQUID) magnetic field intensity analysis was conducted to obtain the hysteresis curves of the nanocarriers (Figure 3e). The saturation magnetization was 75.1 emu/g for Fe_3_O_4_, 33.4 emu/g for MGO, and 5.5 emu/g for MGO-PEG-CET. The reduced Fe_3_O_4_ weight percentage in the nanocarriers may lead to reduced saturation magnetization [50]. Thus, the saturation magnetization of MGO calculated from the saturation magnetization is 44% that of GO, which is close to the values from TGA (42%) and ICP-OES (43%). Further grafting with high molecular weight proteins (avidin molecular weight = ~67 kDa and CET molecular weight = ~152 kDa), and PEG is expected to substantially decrease the weight percentage of Fe_3_O_4_ in MGO-PEG-CET and influence the saturation magnetization. Nonetheless, calculation based on SQUID saturation magnetization indicated that MGO-PEG-CET contained 7% Fe_3_O_4_, which is less than that predicted by ICP-OES (12%). Thus, the reduced saturation magnetization value may originate from the diamagnetic natures of avidin, CET, and PEG on the MGO surface [50,51]. From the magnetization curve, the residue magnetization (remanence) was close to zero without applied external magnetic field (0.13 emu/g for Fe_3_O_4_, 0.6 emu/g for MGO, and 0.1 emu/g for MGO-PEG-CET) and no hysteresis loop was observed. Taken together, MGO-PEG-CET could be confirmed to be superparamagnetic from the SQUID magnetization curve, an important property for magnetic targeted delivery of DOX. 

### 2.2. Drug Loading and Release

**Figure 4:**
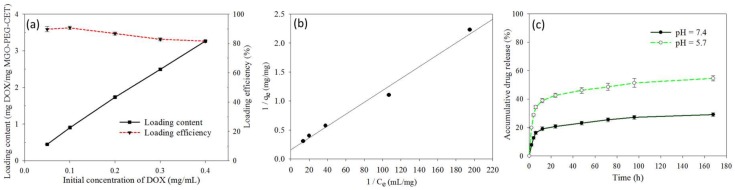
(**a**) Drug loading content and loading efficiency when a DOX solution with different initial concentration was mixed with an equal volume of 0.1 mg/mL MGO-PEG-CET; (**b**) The drug loading was satisfactorily modeled with the Langmuir adsorption isotherm where *C_e_* is the equilibrium DOX concentration in the solution and *q_e_* is the adsorbed DOX amount; and, (**c**) The release of DOX from MGO-PEG-CET/DOX in pH 7.4 and 5.7 PBS at 37 °C.

Figure 4a shows the effect of initial DOX concentration on the loading content (weight of DOX loaded per unit weight of MGO-PEG-CET) and the loading efficiency (weight percentage of DOX loaded) of DOX. As initial DOX concentration increased eight-fold from 0.05 to 0.4 mg/mL, the loading efficiency decreased only slightly from 89.8 to 82.1%, indicating high loading capacity of DOX on MGO-PEG-CET. However, the loading content increased continuously with increasing DOX concentration, in close to linear manner, and reached 3.26 mg/mg. With continuously increasing loading capacity, we also modeled the adsorption DOX to MGO-PEG-CET with the Langmuir adsorption isotherm, assuming MGO-PEG-CET to be an ideal adsorbent composed of series of distinct sites capable of binding DOX. Figure 4b indicated that the adsorption could be modeled satisfactory by the Langmuir adsorption isotherm with (*R*^2^ = 0.99) and the maximal adsorption capacity could be obtained at 6.35 mg/mg [52]. Though initial DOX concentration could be theoretically increased to approach the maximum drug loading, the corresponding increase in solution viscosity hindered recovery of MGO by magnetic separation; therefore, the concentration ratio between MGO-PEG-CET and DOX was set at 1:4 (0.1 mg/mL MGO-PEG-CET with 0.4 mg/mL DOX) for future studies.

The release of DOX from MGO-PEG-CET/DOX was investigated from the cumulative percentage of DOX released at 37 °C in pH 7.4 or pH 5.7 PBS to simulate the extracellular and the intracellular environment (Figure 4c). Sustained release of DOX from MGO-PEG-CET/DOX was observed and the percentage of DOX released at pH 5.7 (54.6%) is about twice that released at pH 7.4 (29%) within one week, thus confirming the pH-sensitive DOX release. Moreover, the initial slope of the release curve at pH 5.7 demonstrates the effective and rapid release of drug in an acidic environment, fulfilling the requirement for intracellular DOX release in the endosome after intracellular uptake [53,54]. GO could adsorb DOX through π–π stacking and by hydrogen bonds between –OH (–COOH) groups of MGO-PEG-CET and –OH group of DOX [55]. Therefore, the pH-sensitive drug release may be due to the weakening of those hydrogen bonds when H^+^ weakens hydrogen bond interactions and promotes drug release. By releasing its drug cargo in the acidic endosomal environment (pH < 6) after intracellular uptake, the cytotoxicity of MGO-PEG-CET/DOX toward cancer cells could be enhanced.

### 2.3. Intracelular Uptake

**Figure 5:**
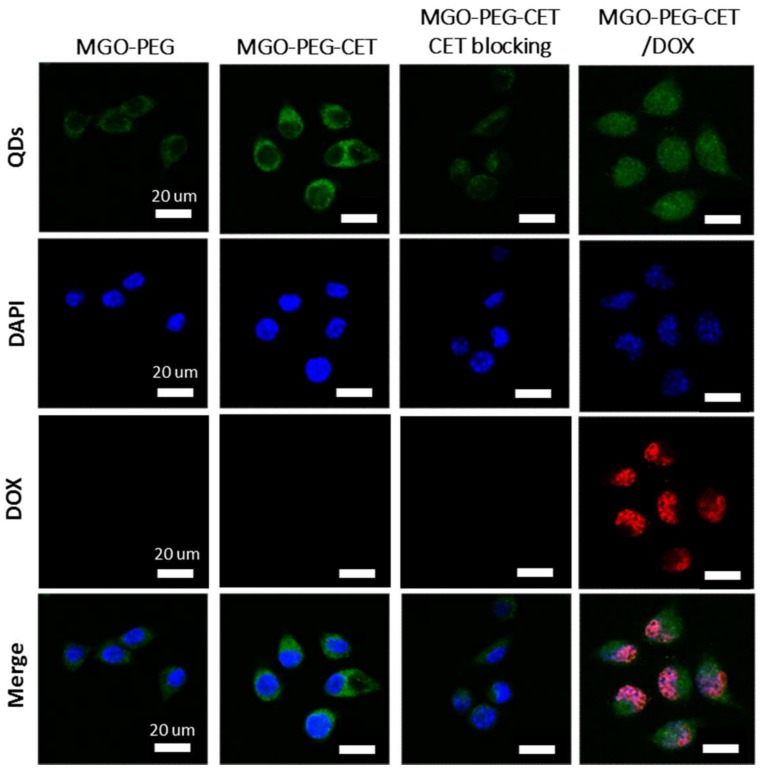
The confocal microscopy images of CT-26 cells after incubated with MGO-PEG, MGO-PEG-CET or MGO-PEG-CET/DOX for 1 h. Blocking of interaction between EGFR and CET (MGO-PEG-CET CET blocking) was carried out by pre-incubating CT-26 cells with CET (1 mg /mL) for 1 h to block EGFR receptors on cell surface followed by incubating with MGO-PEG-CET for 1 h. Bar = 20 µm.

The high expression of EGFR on CT-26 surface is expected to facilitate ligand-targeting of CT-26 cancer cells using CET, an EGFR antibody [56]. This targeting effect could manifest itself through intracellular uptake of quantum dots (QDs)-tagged nanocarriers observed under a confocal microscope. Indeed, the confocal images in Figure 5 confirmed that CT-26 cells showed more intracellular uptake of MGO-PEG-CET than MGO-PEG from the signal intensity of QDs. Furthermore, the intracellular uptake of MGO-PEG-CET was inhibited when CT-26 cells were pre-treated with CET, thus confirming the receptor-mediated endocytosis of MGO-PEG-CET, as EGFR on cell surface could be blocked with excess CET. Therefore, we could confirm the enhanced intracellular uptake of CET-conjugated nanocarrier is governed by the binding of CET in MGO-PEG-CET to EGFR on CT-26 surface, which could provide an active targeting mechanism for targeted drug delivery.

**Figure 6:**
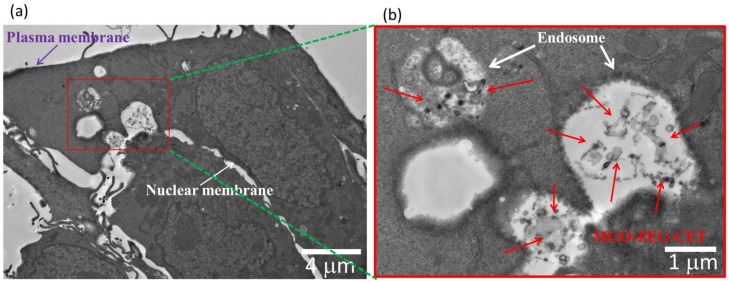
The transmission electron microscope (TEM) micrographs of CT-26 cells after contacting with MGO-PEG-CET for 1 h, bar = 4 µm. (**b**) is the magnified image of the red square shown in (**a**), bar = 1 μm.

The process of endocytosis was further confirmed by TEM. The TEM micrographs in Figure 6 clearly identified aggregates of MGO-PEG-CET within the endosomes after intracellular uptake, which are located in the cell cytoplasm and in close proximity to the cell nucleus. The endocytosis of MGO-PEG-CET is consistent with a previous report that studied the size-dependent intracellular uptake of protein-coated GO [57]. The authors reported that small GO (420 nm equivalent diameter) enter cells mainly through clathrin-mediated endocytosis, while large GO (860 nm equivalent diameter) enter through both phagocytosis and endocytosis.

Visualization of QDs-tagged MGO-PEG-CET/DOX after contacting with CT-26 cells revealed the green fluorescence of QDs-labeled MGO-PEG-CET in the cell cytoplasm, while the red fluorescence of DOX could only be identified in cell nuclei and merged with the blue fluorescence from the DAPI-stained cell nuclei (Figure 5). This implied DOX could be released from MGO-PEG-CET/DOX in the acidic intracellular environment after endocytosis, followed by releasing DOX into the nucleus to chelate with DNA molecules and exert cytotoxicity toward cancer cells [58]. Taken together, the confocal microscopy and TEM results strongly suggested that the cytotoxicity effects of DOX toward CT-26 could be facilitated by endocytosis of MGO-PEG-CET/DOX, followed by drug release at low endosomal pH value to enhance the anticancer activity of the drug.

### 2.4. Magnetic Targeting and Laser-Induced Hyperthermia

**Figure 7:**
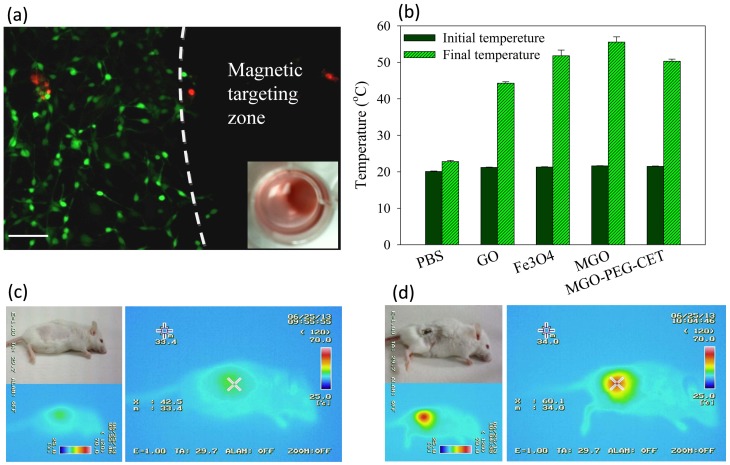
(**a**) Live/Dead staining of CT-26 cells after contacting with MGO-PEG-CET/DOX in a cell culture dish with a magnetic targeting zone created in a well of a culture dish by placing a magnet at the bottom of the well. The insert shows that MGO-PEG-CET/DOX was guided to the right corner of the well in the culture dish. Green: live cells; red: dead cells. Bar = 100 µm; (**b**) Temperature changes in vitro after exposure different nanocarriers (1 mg/mL in 0.2 mL of PBS) to NIR light at 2.5 W/cm^2^ for 3 min. The in vivo temperature rise from thermal imaging of healthy BALB/c mouse (**c**) and tumor-bearing mouse (**d**) after intravenous injection of MGO-PEG-CET (7 mg/kg), followed by guidance with a magnetic field (1400 Gauss) for 2 h and exposure to NIR light (2.5 W/cm^2^ for 5 min).

Through Live/Dead cell viability staining, we verified that an applied magnetic field in vitro could successfully guide MGO-PEG-CET/DOX to the magnetic targeting zone created by a magnet under the well in the cell culture dish. Within the magnetic targeting zone, there were hardly any live cells, while dead cells within this zone were likely to be detached from the well surface and removed during rinsing (Figure 7a). In contrast, abundant live cells (stained green) were detected outside the magnetic targeting zone, endorsing the possibility to magnetically guide MGO-PEG-CET/DOX to the tumor site in vivo using a magnetic field for magnetic targeting [58].

The photothermal effect was studied in vitro by measuring the temperature rise of solutions containing nanocarriers after irradiating with NIR laser at 2.5 W/cm^2^ for 3 min (Figure 7b). Both GO and MGO showed a temperature rise, but the temperature increase was more pronounced for MGO. This is consistent with previous reports that both GO [59] and Fe_3_O_4_ magnetic nanoparticles [60] showed photothermal effects after absorbing light in the NIR region. The introduction of polymer and protein, such as PEG, avidin, and CET, may partially hamper the absorption of NIR light by MGO, resulting in a lower temperature rise for MGO-PEG-CET.

To observe the photothermal effect in vivo, MGO-PEG-CET was injected into healthy BALB/c mice (Figure 7c) or tumor-bearing BALB/c mice with CT-26 cells implanted subcutaneously (Figure 7d). Magnets were attached to the tumor for 2 h and both of the mice were exposed to NIR laser for 5 min at 2.5 W/cm^2^. The regional temperature of the healthy mouse only rose to 42.5 °C, whereas the regional temperature of the tumor-bearing mouse rose to 60.1 °C. These results verified that MGO-PEG-CET is suitable for photothermal therapy in vivo [30].

### 2.5. Biocompatibility of Nanocarriers

**Figure 8:**
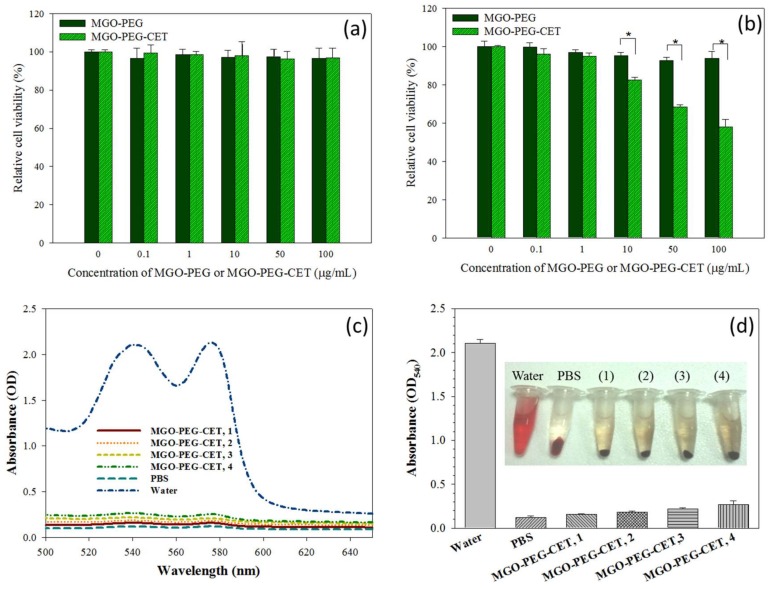
The biocompatibility of MGO-PEG and MGO-PEG-CET toward 3T3 fibroblasts (**a**) and CT-26 (**b**) by incubating cells with nanocarrier at different concentrations for 24 h and the relative cell viability was determined by MTT assays relative to culture medium (* *p* < 0.05); The hematological compatibility of MGO-PEG-CET was determined from the absorption spectra of the supernatant of MGO-PEG-CET after incubation with red blood cells in PBS for 2 h (**c**) for hemolytic assay and the optical density at 540 nm (OD_540_) of the supernatant of MGO-PEG-CET was determined (**d**). Water and PBS were used as the positive and the negative controls, respectively. The temperature was at 37 °C. The concentrations of MGO-PEG-CET were 100 µg/mL (1), 200 µg/mL (2), 400 µg/mL (3) and 800 µg/mL (4) in (**d**).

Biocompatibility tests were performed to assess the safety of the nanocarriers, including the cell viability test and hemolysis assay. The cell viability test was conducted on 3T3 fibroblast and CT-26 cells, which was incubated with different concentrations of MGO-PEG and MGO-PEG-CET. Both MGO-PEG and MGO-PEG-CET were non-toxic to fibroblasts in the concentration studies (Figure 8a). For CT-26, MGO-PEG was also biocompatible up to 100 μg/mL (Figure 8b). In contrast, MGO-PEG-CET shows mild toxicity from 10 to 100 μg/mL, and the relative cell viability was significantly significant different from those of MGO-PEG. It is well-documented that CET causes cytotoxicity to EGFR-expressing cancer cells, which renders CET to be a FDA approved drug for treating colon and head/neck cancers [61,62]. This feature is advantageous when considering that CET in the drug-free nanocarrier (MGO-PEG-CET) may also contribute to the killing of CT-26 cells. 

An in vitro hemolysis assay was conducted to verify blood biocompatibility of MGO-PEG-CET. The absorption spectra (Figure 8c) of the supernatant of MGO-PEG-CET (31.25~250 μg/mL in PBS) after incubation with red blood cells (RBCs) at 37 °C for 2 h were conducted, with deionized (DI) water and PBS being, respectively, designated as the positive and negative controls. The osmotic pressure of DI water caused RBCs to rupture; thus, the absorbance spectrum of the DI water group showed absorbance values significantly higher than that of other groups. No visible hemolysis effect was observed after incubation with MGO-PEG-CET. The solution absorbance at 540 nm (OD_540_) was the lowest for PBS, but slightly increased with increasing MGO-PEG-CET concentration, indicating that the nanocarrier caused slight but acceptable RBC damage (Figure 8d). That both of the solution colors and the absorbance of the MGO-PEG-CET solution were similar to that of the PBS endorsed minimum hemolysis due to MGO-PEG-CET and its safety for in vivo application as a drug carrier.

### 2.6. The Efficacy of Combined Therapy In Vitro and In Vivo

**Figure 9:**
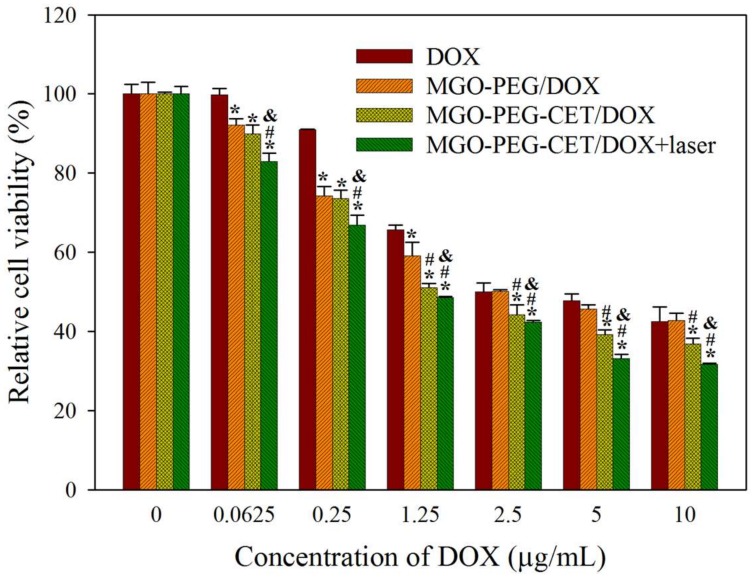
In vitro cytotoxicity of DOX, MGO-PEG/DOX, MGO-PEG-CET/DOX and MGO-PEG-CET/DOX + laser after contacted with CT-26 cells for 24 h. The laser group was subject to 2.5 W/cm^2^ NIR light exposure for 3 min after treating with MGO-PEG-CET/DOX. * *p* < 0.05 compared with DOX. ^#^ *p* < 0.05 compared with MGO-PEG/DOX. ^&^ *p* < 0.05 as compared with MGO-PEG-CET/DOX.

Cell cytotoxicity assay was performed to assess the half maximal inhibitory concentration (IC50) of DOX after 24 h incubation with CT-26 cells in order to compare the efficacy of different treatments in vitro (Figure 9). The IC50 of DOX, MGO-PEG/DOX, MGO-PEG-CET/DOX, and MGO-PEG-CET/DOX + laser were 5.64, 2.64, 1.48, and 1.17 μg/mL, respectively. The results showed that MGO-PEG/DOX showed higher cytotoxicity than DOX, owing to the cellular uptake of MGO-PEG/DOX. The cytotoxicity could be further enhanced using MGO-PEG-CET/DOX with CET ligand-targeting to enhance the intracellular uptake of the nanocarrier and increase intracellular DOX concentration (Figure 5). Most importantly, MGO-PEG-CET/DOX treatment, followed by laser irradiation showed the highest cytotoxicity toward CT-26 cells with only one-fifth the IC50 of DOX, supporting the synergistic effects of chemotherapy and photothermal therapy.

**Figure 10:**
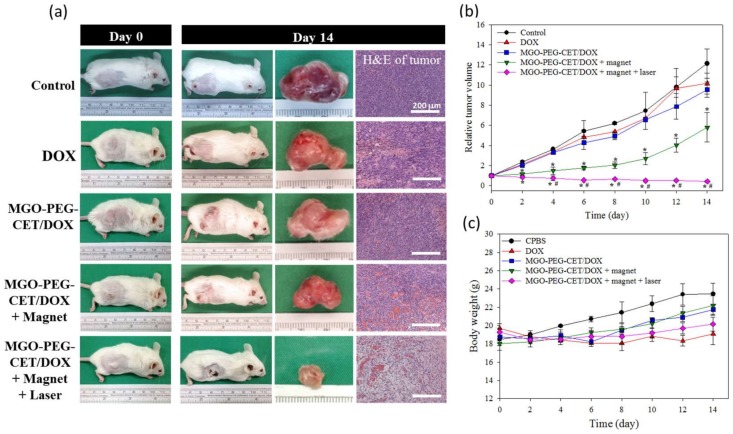
The anti-tumor efficacy in vivo with tumor-bearing BALB/C mice. BALB/c mice were subcutaneously implanted with CT-26 cells and were given different treatment by intravenous injection of normal saline (control), DOX, MGO-PEG-CET/DOX, MGO-PEG-CET/DOX + magnet, and MGO-PEG-CET/DOX + magnet + laser (30 mg/kg DOX). (**a**) The gross observation of tumor-bearing BALB/c mice on day 0 and 14, the gross view of incised tumor and the H&E staining of the incised tumor on day 14 (bar = 200 µm); The relative tumor volume (**b**) and body weight (**c**) were recorded. * *p* < 0.05 compared with control, DOX, and MGO-PEG-CET/DOX, ^#^ *p* < 0.05 as compared with MGO-PEG-CET/DOX + magnet.

The antitumor efficacy of MGO-PEG-CET/DOX was studied in vivo in a xenograft tumor model in mice. BALB/c mice with subcutaneous CT-26 tumors of 60–100 mm^3^ were subjected to treatment with normal saline (control) and DOX in different ways. Gross images of the tumor-bearing mice taken on day 0 and 14 demonstrated the apparent tumor size differences in each group and the tumor removed from the animal on day 14 supported the anti-tumor effects with each treatment, but to a different degree (Figure 10a). Based on results of H&E staining of tumor tissue on day 14, necrosis of the cancer cells was most substantial in MGO-PEG-CET/DOX + magnet and MGO-PEG-CET/DOX + magnet + laser groups [59], but cell growth was observed in control, DOX and MGO-PEG-CET/DOX groups (Figure 10a).

The tumor volumes were recorded every other day up to 14 days and were expressed as relative tumor volume after normalizing the tumor volume at each time point with the tumor volume at day 0. At day 14, the average values of relative tumor volumes were 12.1 (control), 10.1 (free DOX), 9.5 (MGO-PEG-CET/DOX), 5.8 (MGO-PEG-CET/DOX + magnet) and 0.42 (MGO-PEG-CET/DOX + magnet + laser) (Figure 10b). When compared to the control, only MGO-PEG-CET/DOX + magnet and MGO-PEG-CET/DOX + magnet + laser groups showed substantial tumor suppression throughout the observation period (* *p* < 0.05). Although both free DOX and MGO-PEG-CET/DOX groups showed a general trend of tumor volume reduction as compared with the control with MGO-PEG-CET/DOX consistently performed better than DOX, both of the groups did not show significant difference in tumor volume from the control throughout the experiment. This underscores the importance of dual targeting with magnetic guidance in addition to ligand-targeting at the tumor site (MGO-PEG-CET/DOX + magnet) to bring about a significant difference from the control group in tumor volume. Nonetheless, the MGO-PEG-CET/DOX + magnet treatment failed to suppress tumor growth after day 8 with a rapid increase of tumor volume. This uncontrolled increase in tumor size could be alleviated by combining with photothermal therapy using laser light. Indeed, Only the MGO-PEG-CET/DOX + magnet + laser treatment could continuously suppress tumor growth and shrank the tumor size to a value less than the original value throughout the 14-day observation period. Undoubtedly, the significance of local hyperthermia for photothermal tumor ablation could be inferred for this remarkable enhancement in treatment efficacy [63]. As shown in Figure 10c, the mouse’s body weight did not exhibit any significant difference between groups. However, mice in the control group showed a trend of better weight gain when compared to other groups under DOX treatment, which could be ascribed to the common adverse effect from chemotherapy. However, we did not observe any changes in appetite and behavior throughout the observation period for all of the mice under treatment.

## 3. Materials and Methods

### 3.1. Materials

Graphene oxide (N002-PDE) powder was obtained from Angstron Materials Inc. (Dayton, OH, USA). Potassium bromide (KBr) was purchased from Showa Chemical Co. (Tokyo, Japan). 1-(3-dimethylaminopropyl)-3-ethylcarbodiimide hydrochloride (EDC), and *N*-hydroxysuccinimide (NHS) were purchased from Acros Organics (Geel, Belgium). Avidin and cetuximab (CET) was purchased from Calbiochem (San Diego, CA, USA) and Merck (Darmstadt, Germany), respectively. Biotin-PEG-NHS (molecular weight = 3400 Da) was purchased from Nanocs Inc. (New York, NY, USA). Doxorubicin (DOX), 3-(4,5-Dimethyl-2-thiazolyl)-2,5-diphenyl-2H-tetrazolium bromide (MTT), 4′,6-diamidine-2′-phenylindole dihydrochloride (DAPI), and RPMI-1640 medium for cell culture were all purchased from Sigma (St Louis, MO, USA). Live/Dead viability/cytotoxicity kit was purchased from Invitrogen (Carlsbad, CA, USA). Fetal bovine serum (FBS) purchased from Hyclone, GE Healthcare (Logan, UT, USA) was used for cell culture. All of the reagents were of analytical grade. 

### 3.2. Preparation of Magnetic Graphene Oxide (MGO)

GO nano-platelets was prepared by a modified Hummers’ method, as reported before [20]. Chemical co-precipitation was used to deposit Fe_3_O_4_ on GO surface by dispersing 25 mg of GO, 108 mg of FeCl_3_∙6H_2_O and 40 mg of FeCl_2_∙4H_2_O (mole ratio of Fe^2+^:Fe^3+^ = 1:2) in 50 mL of deionized (DI) water by sonication for 30 min. The solution was purged with N_2_ for 30 min (to prevent oxidation of Fe_3_O_4_) and was heated to 65 °C. Next, 1 g of ClCH_2_COOH was added to the solution to convert –OH to –COOH. After 1 h, 2 g of NaOH was added to the solution for reaction at a basic environment for an additional 30 min. A magnet was used to collect magnetic graphene oxide (MGO) from the solution, followed by washing with copious DI water. The amount of –COOH groups on MGO surface was determined by the toluidine blue O (TBO) assays.

### 3.3. Preparation of MGO-PEG-CET and MGO-PEG-CET-QDs

0.05 mL of 60 mM 1-ethyl-3-(3-dimethylaminopropyl) carbodiimide (EDC) and 0.05 mL of 60 mM *N*-hydroxysuccinimide (NHS) were prepared in phosphate-buffered saline (PBS, pH = 7.4) and mixed with 0.5 mL of MGO (1 mg/mL) solution prepared above at 4 °C for 30 min for activation of the –COOH groups of MGO. 1 mg of avidin was then added and the solution was left to react for 12 h at 4 °C for formation of amide bond between –COOH of MGO and –NH_2_ of avidin (MGO-avidin). MGO-avidin was recovered from the solution with a magnet and washed with PBS. The amount of avidin in MGO-avidin was quantified from the amount of unbound avidin in the solution using the Coomassie (Bradford) Protein Assay Kit (Thermo Fisher Scientific, Waltham, MA, USA).

To synthesized biotin-PEG-CET and biotin-PEG-QDs, 0.2 mg of biotin-PEG-NHS was reacted with 4.2 mg of CET or 20 µL of quantum dots (QDs, QSA-490, with amine group from Ocean Nanotech, San Diego, CA, USA) in 1 mL PBS for 12 h at 4 °C through a spontaneous covalent bond formation between NHS esters and –NH_2_ groups in CET or QDs. Biotin-PEG-CET was reacted with MGO-avidin prepared above and incubated at 4 °C for 30 min for binding between avidin and biotin to from MGO-PEG-CET. MGO-PEG-CET was separated from the solution with a magnet and its CET content was determined from the unbound CET in the supernatant using Coomassie (Bradford) Protein Assay Kit. Biotin-PEG-QDs were used to bind empty biotin binding sites of avidin on MGO-PEG-CET to prepare fluorescently labelled MGO-PEG-CET-QDs. MGO-PEG and MGO-PEG-QDs were prepared similarly by replacing CET with glycine to react with biotin-PEG-NHS.

### 3.4. Physico-Chemical Properties of Nanocarriers

The particles size, polydispersity (PDI) and zeta potential of nanocarriers were determined by dynamic light scattering (DLS) using a Nano ZA90 Zetasizer (Malvern Instruments Ltd., Worcestershire, UK) with particle suspensions that were prepared in DI water. For transmission electron microscopy (TEM), the particles was diluted to 0.01 mg/mL in DI water and then dropped onto a 200 mesh carbon-coated copper grid, followed by drying at 25 °C for one day before loading into the microscope. MGO-PEG-CET was stained with 2% phosphotungstic acid for 30 s before drying. The morphology and size of particle were observed by TEM (JEOL JEM-1230, Tokyo, Japan) at 100 kV. For Fourier transform infrared (FTIR) spectroscopy, the samples were blended with KBr, compressed to form a pellet and analyzed with a TENSOR II FTIR Spectrometer (Bruker Optics Inc., Billerica, MA, USA). The transmission spectra were obtained from 400 to 4000 cm^−1^ at 2.5 mm/s with a resolution of 4 cm^−1^. The iron contents of samples were analyzed by inductively coupled plasma optical emission spectroscopy (ICP-OES, Optima 2100 DV, Perkin Elmer, Waltham, MA, USA). For X-ray diffraction (XRD) analysis of the crystal structures of samples, a D2 PHASER X-ray powder diffractometer (Bruker AXS Inc., Madison, WI, USA) was used by scanning dried power in the 2 θ range of 5°–70°. The step size was 0.04° and measurement time was 2 s per step. The phases were compared with the JCPDS database for identification. The crystallite size was determined using the Debye-Scherrer equation. Thermogravimetric analysis (TGA) was conducted with 8~10 mg of powder sample in nitrogen atmosphere from 25 to 750 °C, with a heating rate of 10 °C/min using a Q50 TGA from TA Instruments (New Castle, DE, USA). The magnetization curves were obtained with a superconducting quantum interference device (SQUID) magnetometer (MPMS XL-7, Quantum Design, San Diego, CA, USA) at 25 °C and applied magnetic field of 10,000 G.

### 3.5. Drug Loading and Release

Doxorubicin (DOX) loading onto MGO-PEG-CET was accomplished by mixing 0.1 mg MGO-PEG-CET with different amount of DOX in 1 mL PBS (pH 7.4) at 4 °C for 24 h. After separating MGO-PEG-CET/DOX by a strong magnet, the concentration of DOX in the supernatant was determined by a UV/Vis spectrophotometer (U3010, Hitachi, Tokyo, Japan) at 490 nm. The amount of DOX on MGO-PEG-CET/DOX was calculated from mass balance with the drug loading content (mg/mg) being defined as weight of DOX loaded/weight of MGO-PEG-CET and the drug loading efficiency (%) defined as (weight of DOX loaded/weight of DOX initially added) × 100.

For drug release, MGO-PEG-CET/DOX was placed in 1 mL of PBS (pH 5.7 or pH 7.4) and shaken at 120 rpm and 37 °C in dark. A magnet was used to separate MGO-PEG-CET/DOX at predetermined time points and the precipitate was re-suspended with 1 mL of fresh PBS of the same pH. The amount of DOX released from PEG-MGO-CET/DOX was determined from DOX concentration in the supernatant using a UV/Vis spectrophotometer at 490 nm in a cumulative manner with drug release (%), defined as (weight of DOX released/weight of DOX loaded) × 100.

### 3.6. Intracellular Uptake

CT-26 murine colonic carcinoma cells were obtained from Professor Chia-Rui Shen at the Graduate Institute of Medical Biotechnology of Chang Gung University, Taiwan. To observe the intracellular uptake of nanocarriers by CT-26 cells, 5 × 10^4^ cells were seeded to 15-mm glass slides placed in a 24-well plate. After adding cell culture medium (RPMI-1640 with 10% FBS) and cultured for 24 h, MGO-PEG-QDs, MGO-PEG-CET-QDs, or MGO-PEG-CET-QDs/DOX was separately added to each well and incubated for another 1 h. After removing the cell culture medium and washing with PBS, cells was fixed with 4% paraformaldehyde for 15 min and were stained with 1 μg/mL DAPI for 10 min. To further confirm that the interaction between CET and EGFR molecules was the mechanism for enhancing the targeting efficacy of CET-conjugated nanocarriers, CT-26 cells were pre-treated with 1 mg/mL of CET for 1 h to block the EGFR molecules on cell surface before incubating with MGO-PEG-CET-QDs. The slides were observed under a confocal laser scanning microscope (Zeiss LSM 510 Meta, Oberkochen, Germany). The uptake of MGO and release of DOX could be visualized by the green fluorescence of QDs-labelled MGO-PEG or MGO-PEG-CET and the red fluorescence of DOX. The excitation wavelength is Red/Green/Blue = 543/488/364 nm and the emission wavelength is Red/Green/Blue = 550–650/500–550/407–482 nm.

The phenomenon of cell uptake was also observed through transmission electron microscope (TEM), where 5 × 10^4^ CT-26 cells were grown on ThermoNox (Nunc, Thermo Fisher Scientific, Waltham, MA, USA) coverslips and were treated with MGO-PEG-CET for 24 h. After fixing with a mixture of 2% glutaraldehyde and 2.5% paraformaldehyde for 2 h, 0.1 M sodium cacodylate buffer (pH 7.4) was used to rinse the cells followed by post-fixing 30 min in 1.0% osmium tetroxide. Graded ethanol series (30%, 50%, 70%, 80%, 95%, and 100%) were used to dehydrate the cells for 10 min at each concentration, followed by two rinses in 100% propylene oxide. With infiltration and embedding in epoxy resins for 48 h at 60 °C, ~80 nm ultrathin specimen were sectioned and examined under a FEI/Philips CM 120 TEM (FEI, Hillsboro, OR, USA).

### 3.7. Photothermla Effect

In vitro photothermal effect was determined in 0.2 mL suspensions of GO, MGO, and MGO-PEG-PET (1 mg/mL in PBS) in 96-well cell culture plates by irradiating with an 808 nm continuous-wave near infrared (NIR) laser at a power of 2.5 W/cm^2^ (0.4 cm^2^ laser area) for 3 min. The temperature of the solution before and after exposure to NIR light was determined with a K-Type thermal couple thermometer (Hanna Instruments, Woonsocket, RI, USA).

In vivo photothermal effect was from thermal imaging of a tumor-bearing BALB/c mouse after the intravenous injection of MGO-PEG-CET. The tumor-bearing mouse was injected with MGO-PEG-CET (7 mg/kg), followed by guidance with a magnetic field (1400 Gauss) for 2 h and exposure to NIR light at 2.5 W/cm^2^ (0.4 cm^2^ laser area) for 5 min. Thermal images were captured with an infrared thermal imaging camera (Thermo GEAR G100EX, Avio, Tokyo, Japan) to measure the temperature distribution around the tumor area. A healthy BALB/c mouse was used as a control after exposure to the same NIR light for 5 min at similar same location.

### 3.8. Magnetic Guidance In Vitro

To examine the effect of magnetic guidance of MGO-PEG-CET, 0.5 mL cell culture medium containing CT-26 cells (5 × 10^4^) was added to each well of a 24-well plate and was cultured for 24 h. After adding MGO-PEG-CET/DOX to each well to reach a final concentration of 0.1 mg/mL, the culture plate was placed on a magnetic separator, which had 7.5 mm diameter permanent magnets glued to the center of each well, followed by 24 h cell culture. After washing with PBS, a Live/Dead cell viability assay was conducted by examining with an inverted fluorescence microscope to determine live and dead cells around the magnetic targeting zone created by the magnet.

### 3.9. Blood Compatibility Analysis

The hemolysis assay was conducted to evaluate the whole blood compatibility of MGO-PEG-CET. The red blood cells (RBCs) from Sprague-Dawley (SD) rats (BioLASCO, Taipei, Taiwan) were obtained by removing the serum from the whole blood after centrifugation at 3500 rpm at 4 °C for 10 min. All of the animal experiments were conducted according to protocols approved by the Chang Gung University’s Institutional Animal Care and Use Committee (IACUC Approval No.: CGU15-168). Following wash with PBS five times, the cells were diluted to ten times of the original volume with PBS. The diluted RBC (0.3 mL) suspension was mixed with 1.2 mL of DI water (positive control), 1.2 mL of PBS (negative control), or 1.2 mL of different concentration of MGO-PEG-CET in PBS. The mixtures were incubated at 37 °C for 30 min and centrifuged. The absorbance values of the supernatants were recorded from 500 to 650 nm using an ultraviolet-visible (UV/Vis) Spectrophotometer and compared at 540 nm (OD_540_) for all the samples. 

### 3.10. In Vitro Cytotoxicity

Approximately 5 × 10^3^ CT-26 cells in 200 µL cell culture medium were placed in each well of a 96-well culture plate and cultured for 24 h. After removing the spent culture medium, different concentrations of DOX (free DOX, MGO-PEG/DOX, or MOG-PEG-CET/DOX) in 200 µL cell culture medium was added to each well and incubated at 37 °C for 24 h. The medium in each well was removed and each well was washed with PBS, followed by adding 200 μL diluted MTT solution (1 mg/mL in culture medium) and incubated for 2 h at 37 °C in dark. The MTT solution was removed and purple formazan crystals in each well was dissolved with 200 µL dimethyl sulfoxide and the solution absorbance was measured with a microplate reader at 570 nm (OD_570_). The cytotoxicity to CT-26 cells was determined from the relative cell viability (%) relative to cells cultured in cell culture medium.

### 3.11. Mouse Subcutaneous Tumor Model

Female BALB/c mice weighing approximately 15–20 g (4–6 weeks old) were purchased from BioLASCO (Taipei, Taiwan). All of the animal experiments were conducted according to protocols that were approved by the Chang Gung University’s Institutional Animal Care and Use Committee (IACUC Approval No.: CGU15-168). CT-26 cells were harvested by 0.1% trypsin-EDTA, washed once with PBS, re-suspended in serum-free RPMI-1640 (1 × 10^7^ cells in 100 µL) and were subcutaneously injected into the right flank of each mouse. When the tumors had grown to approximately 60–100 mm^3^ (about 14 days), the tumor-bearing mice were randomized into five groups (*n* = 6 in each group): group 1 (control) received 200 µL intravenous (IV) injection of normal saline; group 2 received 200 µL IV injection of DOX solution (30 mg/kg); group 3 received 200 µL IV injection of 9.2 mg/kg MGO-PEG-CET/DOX (containing 30 mg/kg DOX); group 4 was treated as in groups 3, but the tumor was exposed to a magnetic field of 1400 Gauss with a magnet for 2 h after IV injection; group 5 was treated as in group 4 but the tumor was exposed to additional NIR irradiation (808 nm wavelength, 2.5 W/cm^2^) for 5 min every two days. Injections were carried out over 2 min through the tail vein, with withdrawal of needle over 1 min to prevent back leak. The animal body weight and tumor volume were continuously monitored on alternate days for two weeks post treatment [64]. For ethical reasons, animals were euthanized when the volume of the implanted tumor reached 2 cm^3^. The tumor size was measured using a caliper and defined as: tumor volume = (length × width × width)/2. Tumors were collected on day 14 of the treatment, fixed in 10% buffered formalin, followed by paraffin-embedment and sectioning to 2–3 μm thickness for hematoxylin and eosin (H&E) staining.

### 3.12. Statistical Analyses

All data were reported as mean ± standard deviation (SD) and subject to one-way analysis of variance (ANOVA) analysis. Tukey’s post-hoc test was used to determine the difference between any two groups with *p*-value < 0.05 considered to be statistically significant.

## 4. Conclusions

We presented a dual-targeting nanomedicine approach for cancer therapy using MGO-PEG-CET/DOX that combines co-precipitated Fe_3_O_4_ magnetic nanoparticles and CET (an EGFR antibody) for magnetic and receptor-mediated ligand-targeting of malignant CT-26 mouse colon carcinoma. The combinatory chemo-photothermal therapy comprises the antitumor drug DOX and laser-induced hyperthermia with contribution from the EGFR-specific antibody (CET). Through this comprehensive design, the MGO-PEG-CET/DOX showed enhanced cytotoxicity toward CT-26 in vitro and inhibited tumor propagation in vivo. The anti-tumor effects could be augmented using an NIR laser for photothermal therapy in vitro and in vivo. From the proof-of-concept report using magnetic targeting plus NIR laser irradiation, which successfully shrunk tumors to 42% of its original volume in 14 days, this study provides a new paradigm to evolve traditional chemotherapeutic drug (DOX) and to overcome its side effects. The dual targeting MGO-PEG-CET drug delivery system could be implied to offer an extraordinary platform in promoting the success of cancer therapy.

## Figures and Tables

**Table 1:** The particle size and polydispersity index (PDI) by dynamic light scattering (DLS) and zeta potential of different nanocarriers.

Sample	Particle Size (nm)	Polydispersity Index	Zeta Potential (mV)
GO	136.4 ± 5.7	0.18 ± 0.05	−44.3 ± 2.8
MGO	205.5 ± 19.9 *	0.28 ± 0.03 *	−35.1 ± 0.9 *
MGO-PEG-CET	215.7 ± 18.4 *	0.29 ± 0.03 *	−19.8 ± 0.4 *^,#^

* *p* < 0.05 compared with GO; ^#^ *p* < 0.05 compared with MGO.

## References

[1] Organization W.H. Cancer. http://www.who.int/cancer/en/.

[2] Feng S.S., Chien S. (2003). Chemotherapeutic engineering: Application and further development of chemical engineering principles for chemotherapy of cancer and other diseases. Chem. Eng. Sci..

[3] Skalickova S., Loffelmann M., Gargulak M., Kepinska M., Docekalova M., Uhlirova D., Stankova M., Fernandez C., Milnerowicz H., Ruttkay-Nedecky B. (2017). Zinc-modified nanotransporter of doxorubicin for targeted prostate cancer delivery. Nanomaterials.

[4] Au J.L., Jang S.H., Zheng J., Chen C.T., Song S., Hu L., Wientjes M.G. (2001). Determinants of drug delivery and transport to solid tumors. J. Controll. Release.

[5] Lammers T., Aime S., Hennink W.E., Storm G., Kiessling F. (2011). Theranostic nanomedicine. Acc. Chem. Res..

[6] Quader S., Kataoka K. (2017). Nanomaterial-enabled cancer therapy. Mol. Ther..

[7] Goldberg M., Langer R., Jia X. (2007). Nanostructured materials for applications in drug delivery and tissue engineering. J. Biomater. Sci. Polym. Ed..

[8] Duncan R. (2003). The dawning era of polymer therapeutics. Nat. Rev. Drug Discov..

[9] Maeda H. (2001). The enhanced permeability and retention (EPR) effect in tumor vasculature: The key role of tumor-selective macromolecular drug targeting. Adv. Enzyme Regul..

[10] Matsumura Y., Maeda H. (1986). A new concept for macromolecular therapeutics in cancer chemotherapy: Mechanism of tumoritropic accumulation of proteins and the antitumor agent smancs. Cancer Res..

[11] Lu Y.J., Wei K.C., Ma C.C., Yang S.Y., Chen J.P. (2012). Dual targeted delivery of doxorubicin to cancer cells using folate-conjugated magnetic multi-walled carbon nanotubes. Colloids Surf. B Biointerfaces.

[12] McCallion C., Burthem J., Rees-Unwin K., Golovanov A., Pluen A. (2016). Graphene in therapeutics delivery: Problems, solutions and future opportunities. Eur. J. Pharm. Biopharm..

[13] Hong S., Leroueil P.R., Majoros I.J., Orr B.G., Baker J.R., Holl M.M.B. (2007). The binding avidity of a nanoparticle-based multivalent targeted drug delivery platform. Chem. Biol..

[14] Montet X., Funovics M., Montet-Abou K., Weissleder R., Josephson L. (2006). Multivalent effects of rgd peptides obtained by nanoparticle display. J. Med. Chem..

[15] Mejias R., Perez-Yague S., Gutierrez L., Cabrera L.I., Spada R., Acedo P., Serna C.J., Lazaro F.J., Villanueva A., Morales M.P. (2011). Dimercaptosuccinic acid-coated magnetite nanoparticles for magnetically guided in vivo delivery of interferon gamma for cancer immunotherapy. Biomaterials.

[16] Sanson C., Diou O., Thevenot J., Ibarboure E., Soum A., Brulet A., Miraux S., Thiaudiere E., Tan S., Brisson A. (2011). Doxorubicin loaded magnetic polymersomes: Theranostic nanocarriers for mr imaging and magneto-chemotherapy. ACS Nano.

[17] Yang C.L., Chen J.P., Wei K.C., Chen J.Y., Huang C.W., Liao Z.X. (2017). Release of doxorubicin by a folate-grafted, chitosan-coated magnetic nanoparticle. Nanomaterials.

[18] Shang N.G., Papakonstantinou P., McMullan M., Chu M., Stamboulis A., Potenza A., Dhesi S.S., Marchetto H. (2008). Catalyst-free efficient growth, orientation and biosensing properties of multilayer graphene nanoflake films with sharp edge planes. Adv. Funct. Mater..

[19] Yang X.Y., Zhang X.Y., Ma Y.F., Huang Y., Wang Y.S., Chen Y.S. (2009). Superparamagnetic graphene oxide-Fe_3_O_4_ nanoparticles hybrid for controlled targeted drug carriers. J. Mater. Chem..

[20] Sun X., Liu Z., Welsher K., Robinson J.T., Goodwin A., Zaric S., Dai H. (2008). Nano-graphene oxide for cellular imaging and drug delivery. Nano. Res..

[21] Liu J., Cui L., Losic D. (2013). Graphene and graphene oxide as new nanocarriers for drug delivery applications. Act. Biomater..

[22] Li Y.M., Jiang T., Lv Y., Wu Y., He F., Zhuo R.X. (2015). Amphiphilic copolymers with pendent carboxyl groups for high-efficiency loading and controlled release of doxorubicin. Colloids Surf. B Biointerfaces.

[23] Felber A.E., Dufresne M.H., Leroux J.C. (2012). Ph-sensitive vesicles, polymeric micelles, and nanospheres prepared with polycarboxylates. Adv. Drug Deliv. Rev..

[24] Simoes S., Moreira J.N., Fonseca C., Duzgunes N., de Lima M.C. (2004). On the formulation of ph-sensitive liposomes with long circulation times. Adv. Drug Deliv. Rev..

[25] Yan T., Zhang H., Huang D., Feng S., Fujita M., Gao X.D. (2017). Chitosan-functionalized graphene oxide as a potential immunoadjuvant. Nanomaterials.

[26] Seabra A.B., Paula A.J., de Lima R., Alves O.L., Durán N. (2014). Nanotoxicity of graphene and graphene oxide. Chem. Res. Toxicol..

[27] Huang J., Zong C., Shen H., Liu M., Chen B., Ren B., Zhang Z. (2012). Mechanism of cellular uptake of graphene oxide studied by surface-enhanced raman spectroscopy. Small.

[28] Yue H., Wei W., Yue Z., Wang B., Luo N., Gao Y., Ma D., Ma G., Su Z. (2012). The role of the lateral dimension of graphene oxide in the regulation of cellular responses. Biomaterials.

[29] Yang K., Wan J.M., Zhang S., Tian B., Zhang Y.J., Liu Z. (2012). The influence of surface chemistry and size of nanoscale graphene oxide on photothermal therapy of cancer using ultra-low laser power. Biomaterials.

[30] Zhang W., Guo Z., Huang D., Liu Z., Guo X., Zhong H. (2011). Synergistic effect of chemo-photothermal therapy using pegylated graphene oxide. Biomaterials.

[31] Ma X.X., Tao H.Q., Yang K., Feng L.Z., Cheng L., Shi X.Z., Li Y.G., Guo L., Liu Z. (2012). A functionalized graphene oxide-iron oxide nanocomposite for magnetically targeted drug delivery, photothermal therapy, and magnetic resonance imaging. Nano Res..

[32] Huang Y.S., Lu Y.J., Chen J.P. (2017). Magnetic graphene oxide as a carrier for targeted delivery of chemotherapy drugs in cancer therapy. J. Magn. Magn. Mater..

[33] Park J.W., Mok H., Park T.G. (2008). Epidermal growth factor (EGF) receptor targeted delivery of pegylated adenovirus. Biochem. Bioph. Res. Commun..

[34] Jokerst J.V., Lobovkina T., Zare R.N., Gambhir S.S. (2011). Nanoparticle pegylation for imaging and therapy. Nanomedicine.

[35] Gref R., Minamitake Y., Peracchia M.T., Trubetskoy V., Torchilin V., Langer R. (1994). Biodegradable long-circulating polymeric nanospheres. Science.

[36] Höög J.L., Gluenz E., Vaughan S., Gull K., Müller-Reichert T. (2010). Chapter 8—Ultrastructural investigation methods for trypanosoma brucei. Methods in Cell Biology.

[37] Jokar S., Pourjavadi A., Adeli M. (2014). Albumin-graphene oxide conjugates; carriers for anticancer drugs. RSC Adv..

[38] Acharya S., Sahoo S.K. (2011). PLGA nanoparticles containing various anticancer agents and tumour delivery by epr effect. Adv. Drug Deliv. Rev..

[39] Tseng S.H., Chou M.Y., Chu I.M. (2015). Cetuximab-conjugated iron oxide nanoparticles for cancer imaging and therapy. Int. J. Nanomed..

[40] Mahmoud W.E. (2011). Morphology and physical properties of poly(ethylene oxide) loaded graphene nanocomposites prepared by two different techniques. Eur. Polym. J..

[41] Murugan A.V., Muraliganth T., Manthiram A. (2009). Rapid, facile microwave-solvothermal synthesis of graphene nanosheets and their polyaniline nanocomposites for energy strorage. Chem. Mater..

[42] Liang R.P., Liu C.M., Meng X.Y., Wang J.W., Qiu J.D. (2012). A novel open-tubular capillary electrochromatography using beta-cyclodextrin functionalized graphene oxide-magnetic nanocomposites as tunable stationary phase. J. Chromatogr. A.

[43] He H., Gao C. (2010). Supraparamagnetic, conductive, and processable multifunctional graphene nanosheets coated with high-density Fe_3_O_4_ nanoparticles. ACS Appl. Mater. Interfaces.

[44] Zhu Y., Murali S., Cai W., Li X., Suk J.W., Potts J.R., Ruoff R.S. (2010). Graphene and graphene oxide: Synthesis, properties, and applications. Adv. Mater..

[45] Kolhe P., Kannan R.M. (2003). Improvement in ductility of chitosan through blending and copolymerization with peg:  Ftir investigation of molecular interactions. Biomacromolecules.

[46] Shen J.F., Shi M., Ma H.W., Yan B., Li N., Ye M.X. (2011). Hydrothermal synthesis of magnetic reduced graphene oxide sheets. Mater. Res. Bull..

[47] Xu L.Q., Wang L., Zhang B., Lim C.H., Chen Y., Neoh K.G., Kang E.T., Fu G.D. (2011). Functionalization of reduced graphene oxide nanosheets via stacking interactions with the fluorescent and water-soluble perylene bisimide-containing polymers. Polymer.

[48] Ghosh S., Badruddoza A.Z.M., Hidajat K., Uddin M.S. (2013). Adsorptive removal of emerging contaminants from water using superparamagnetic Fe_3_O_4_ nanoparticles bearing aminated beta-cyclodextrin. J. Environ. Chem. Eng..

[49] Wang C., Feng L., Yang H., Xin G., Li W., Zheng J., Tian W., Li X. (2012). Graphene oxide stabilized polyethylene glycol for heat storage. Phys. Chem. Chem. Phys..

[50] Chen J.P., Yang P.C., Ma Y.H., Tu S.J., Lu Y.J. (2012). Targeted delivery of tissue plasminogen activator by binding to silica-coated magnetic nanoparticle. Int. J. Nanomed..

[51] Hsu H.L., Chen J.P. (2017). Preparation of thermosensitive magnetic liposome encapsulated recombinant tissue plasminogen activator for targeted thrombolysis. J. Magn. Magn. Mater..

[52] Wang Y., Yang S.T., Wang Y., Liu Y., Wang H. (2012). Adsorption and desorption of doxorubicin on oxidized carbon nanotubes. Colloids Surf. B Biointerfaces.

[53] Oishi M., Hayashi H., Iijima M., Nagasaki Y. (2007). Endosomal release and intracellular delivery of anticancer drugs using ph-sensitive PEGylated nanogels. J. Mater. Chem..

[54] Lee M., Jeong J., Kim D. (2015). Intracellular uptake and ph-dependent release of doxorubicin from the self-assembled micelles based on amphiphilic polyaspartamide graft copolymers. Biomacromolecules.

[55] Zhang R.Y., Olin H. (2012). Carbon nanomaterials as drug carriers: Real time drug release investigation. Mater. Sci. Eng. C Mater. Biol. Appl..

[56] Cai W., Chen K., He L., Cao Q., Koong A., Chen X. (2007). Quantitative PET of EGFR expression in xenograft-bearing mice using ^64^Cu-labeled cetuximab, a chimeric anti-egfr monoclonal antibody. Eur. J. Nucl. Med. Mol. Imaging.

[57] Mu Q., Su G., Li L., Gilbertson B.O., Yu L.H., Zhang Q., Sun Y.P., Yan B. (2012). Size-dependent cell uptake of protein-coated graphene oxide nanosheets. ACS Appl. Mater. Interfaces.

[58] Chertok B., David A.E., Yang V.C. (2010). Polyethyleneimine-modified iron oxide nanoparticles for brain tumor drug delivery using magnetic targeting and intra-carotid administration. Biomaterials.

[59] Yang K., Zhang S., Zhang G., Sun X., Lee S.-T., Liu Z. (2010). Graphene in mice: Ultrahigh in vivo tumor uptake and efficient photothermal therapy. Nano Lett..

[60] Chu M., Shao Y., Peng J., Dai X., Li H., Wu Q., Shi D. (2013). Near-infrared laser light mediated cancer therapy by photothermal effect of Fe_3_O_4_ magnetic nanoparticles. Biomaterials.

[61] Maiello E., Gebbia V., Manzione L., Giuliani F., Morelli F., Arcara C., Grimaldi A., Colucci G. (2008). Clinical results of EGFR-targeted therapies in advancedcolorectal cancer. EJC Suppl..

[62] Rivera F., Vega-Villegas M.E., Lopez-Brea M.F. (2008). Cetuximab, its clinical use and future perspectives. Anticancer Drugs.

[63] O’Neal D.P., Hirsch L.R., Halas N.J., Payne J.D., West J.L. (2004). Photo-thermal tumor ablation in mice using near infrared-absorbing nanoparticles. Cancer Lett..

[64] Kirui D.K., Khalidov I., Wang Y., Batt C.A. (2013). Targeted near-IR hybrid magnetic nanoparticles for in vivo cancer therapy and imaging. Nanomedicine.

